# [Retracted] MicroRNA‑21 promotes migration and invasion of glioma cells via activation of Sox2 and β‑catenin signaling

**DOI:** 10.3892/mmr.2023.12982

**Published:** 2023-03-22

**Authors:** Guoxuan Luo, Wentao Luo, Xiaohui Sun, Jinzhi Lin, Mo Wang, Yang Zhang, Weishi Luo, Yong Zhang

Mol Med Rep 15: 187–193, 2017; DOI: 10.3892/mmr.2016.5971

Following the publication of the above paper, a concerned reader drew to the Editor's attention that there were several data panels showing the results of Transwell migration and invasion assay experiments in Figs. 1A and 2A that contained overlapping sections of data, such that these data, which were intended to have shown the results from differently performed experiments, appeared to have been derived from a smaller number of original sources. Furthermore, the data panels shown in Fig. 3A for the ‘Control/U343’ and ‘Control/172’, and the ‘miR-21/β-catenin’ and ‘Control/T98’, experiments were also found to be unexpectedly similar, given that these were likewise intended to show the results from differently performed experiments.

After having conducted an independent investigation in the Editorial Office, the Editor of *Molecular Medicine Reports* has determined that the above paper should be retracted from the Journal on account of a lack of confidence in the presented data. The authors were asked for an explanation to account for these concerns, but the Editorial Office did not receive a satisfactory reply. The Editor regrets any inconvenience that has been caused to the readership of the Journal.

